# Individual mRNA expression profiles reveal the effects of specific microRNAs

**DOI:** 10.1186/gb-2008-9-5-r82

**Published:** 2008-05-16

**Authors:** Amit Arora, David AC Simpson

**Affiliations:** 1 Queen's University Belfast, Centre for Vision Sciences, Institute of Clinical Science, Royal Victoria Hospital, Belfast BT12 6BA, UK

## Abstract

**Background:**

MicroRNAs (miRNAs) are oligoribonucleotides with an important role in regulation of gene expression at the level of translation. Despite imperfect target complementarity, they can also significantly reduce mRNA levels. The validity of miRNA target gene predictions is difficult to assess at the protein level. We sought, therefore, to determine whether a general lowering of predicted target gene mRNA expression by endogenous miRNAs was detectable within microarray gene expression profiles.

**Results:**

The target gene sets predicted for each miRNA were mapped onto known gene expression data from a range of tissues. Whether considering mean absolute target gene expression, rank sum tests or 'ranked ratios', many miRNAs with significantly reduced target gene expression corresponded to those known to be expressed in the cognate tissue. Expression levels of miRNAs with reduced target mRNA levels were higher than those of miRNAs with no detectable effect on mRNA expression. Analysis of microarray data gathered after artificial perturbation of expression of a specific miRNA confirmed the predicted increase or decrease in influence of the altered miRNA upon mRNA levels. Strongest associations were observed with targets predicted by TargetScan.

**Conclusion:**

We have demonstrated that the effect of a miRNA on its target mRNAs' levels can be measured within a single gene expression profile. This emphasizes the extent of this mode of regulation *in vivo *and confirms that many of the predicted miRNA-mRNA interactions are correct. The success of this approach has revealed the vast potential for extracting information about miRNA function from gene expression profiles.

## Abstract 2

The effect of a microRNA on the levels of its target mRNAs can be measured within a single gene expression profile

## Background

MicroRNAs (miRNAs) are short oligonucleotides (approximately 22 bp) that regulate gene expression. Target genes are determined by sequence complementarity between the 3' untranslated region (UTR) and the mature miRNA, particularly in a 6 bp 'seed' region [1,2]. A range of algorithms have been developed to predict the genes targeted by specific miRNAs [3]. For example, 'TargetScan' [4,5] searches for conserved 8-mer and 7-mer sites in 3' UTRs that match the seed region of a known miRNA. It is possible, therefore, to obtain lists of the potential target mRNAs for each miRNA. Plant miRNAs, which are often perfectly matched to their target sequences, act primarily by directing mRNA cleavage and degradation [6,7]. In contrast, animal miRNAs have been shown to exert their effect largely via post-transcriptional inhibition of protein synthesis [8]. However, it has been shown that miRNAs expressed in animal cells can affect mRNA levels, not only when they share almost complete complementarity with their target site [9], but more generally when base-pairing is partial [10-12]. When, for example, miR-124, which is known to be characteristic of neuronal tissue, was overexpressed, the genes that were down-regulated at the mRNA level included a preponderance of those expressed at lower levels in neuronal compared to other tissues [11]. Conversely, silencing of miR-122 with a complementary, single-stranded RNA analogue, or 'antagomir', resulted in increased expression of mRNAs that were enriched in miR-122 recognition motifs [13] and miR-122 can direct cleavage of a reporter gene. Depletion of proteins required for miRNA processing has been shown to cause widespread alteration in mRNA levels [14,15].

The suggestion that miRNAs can affect mRNA levels led to the prediction that a miRNA expressed at a high level in a specific tissue might leave a signature on the mRNA expression profile. Sood *et al*. [16] and Farh *et al*. [17] demonstrated that the predicted target genes of known tissue-specific miRNAs (for example, miR-122 in liver; miR-1 in heart/skeletal muscle and miR-7 in pituitary) were expressed at significantly lower levels, as determined by microarray analysis, in their cognate tissue relative to all other tissues.

The conclusive demonstrations that miRNAs can alter mRNA levels suggested to us that, within a specific tissue, the expression of genes predicted to be targeted by a specific mature miRNA might have a detectable inverse relationship with the expression level of that miRNA. This approach has been made feasible by advances in microarray technology and provision of comprehensive gene coverage, which have made global gene expression data increasingly reliable and reproducible [18,19]. Concomitantly, public repositories such as Gene Expression Omnibus (GEO) [20,21] and ArrayExpress [22] have made data from a huge range of tissues available to the scientific community. A method for extracting miRNA signatures from an mRNA expression dataset would be invaluable because it could immediately be applied to analyze miRNA activity in any situation for which microarray gene expression data are available.

Others have had limited success in detecting a significant effect of miRNAs within a single gene expression profile using a non-parametric approach based on gene expression ranking [16]. However, by employing different predicted target and control datasets we were able to observe significant miRNA effects using a similar approach and by direct analysis of absolute target gene expression values (by the term 'target gene expression' we refer to the expression of predicted target genes). We were able to predict many of the previously characterized, highly expressed and/or tissue-specific miRNAs (for example, 14 of 25 in brain). This approach will facilitate investigation of the activity of miRNAs upon mRNA expression, without the need for ranking gene expression of each gene across a series of tissues [11,23].

## Results and discussion

### Detection of miRNA signatures within endogenous gene expression profiles

miRNAs can down-regulate target mRNAs; therefore, one would expect that the target genes of a highly expressed miRNA might be expressed at a significantly lower level than those of a lowly expressed miRNA. In this case it might be possible to detect the presence of miRNAs from the relative expression of their predicted target genes. The profile of miRNAs expressed in one tissue differs from that in another and to test whether different 'signatures' were detectable, we first downloaded mRNA expression profiles for a range of tissues from GEO [20,21]. A range of algorithms have been developed to predict miRNA target genes [3]. However, the scarcity of experimentally confirmed interactions has made it difficult to develop reliable algorithms and validate existing methods. The relevance of existing rules is uncertain [24] and additional factors such as co-factor binding and relative positions of target sites [25,26] undoubtedly play a role. Of the publicly available algorithms, we chose to initially use TargetScan [27] because its requirement for a perfect match to the seed region and cross-species conservation reduce the false-positive rate [3-5]. The resulting higher specificity of this algorithm maximizes the ability to detect effects on expression of real miRNA target genes. After detecting a signal we subsequently tested alternative miRNA target gene prediction algorithms (see below). For every mRNA expression dataset, the mRNA expression of predicted targets was mapped onto the respective miRNA families. The average number of predicted target genes for a single miRNA expressed in a given tissue was 134 (± 9 standard error; the number of predicted target genes for each miRNA expressed in all tissues is shown in Additional data file 1). We then tested the ability of three analytical approaches to detect the effects of variable endogenous miRNA expression on mRNA levels.

### Wilcoxon rank sum test

**Figure 1:**
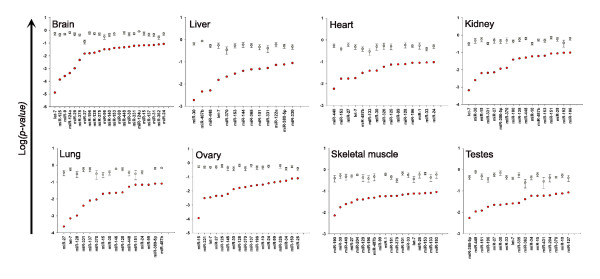
miRNAs with significantly low target gene expression determined by the Wilcoxon rank sum test. The probabilities (log_10_, x-axis for all miRNAs with *p *< 0.1 are plotted in ascending order with red circles for all eight tissues analyzed. For each miRNA the mean probability (± standard error) derived from five random sets of predicted target genes is plotted in grey.

Our first analysis followed the 'tissue-centric' approach described by Sood and colleagues [16]. A vector of expression values for each set of specific miRNA target genes was compared to a vector of expression values for all predicted target genes. For all tissues, miRNAs with significantly low target gene expression were detected (Wilcoxon rank sum test), with lowest *p*-values ranging from 1.29 × 10^-5 ^in brain to 7.23 × 10^-3 ^in skeletal muscle. The results for all tissues are shown in Figure 1. It is notable that well characterized tissue-specific miRNAs, such as miR-122 in liver and miR-124 in brain, are all detected in the expected tissue and not elsewhere. This suggests that the 'signature' that a miRNA exerts upon mRNA expression can be detected within a single gene expression profile, without relation to levels in other tissues as previously reported [16].

### Ranked ratio

**Figure 2:**
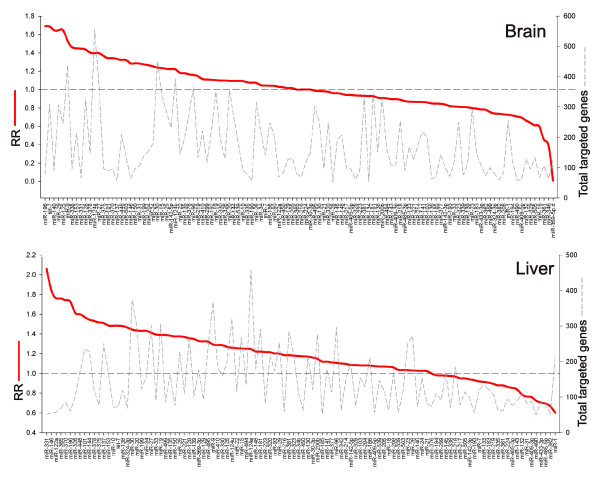
Ranked ratio values for all miRNAs in brain and liver. The miRNAs are ordered by RR values (left-hand y-axis), which are displayed as a red line. The higher values reflect lower expression of predicted target genes and are, therefore, indicative of miRNA activity. The numbers of genes predicted to be targeted by each miRNA (right-hand y-axis) are indicated by the dashed line. Known neural- (for example, miR-29) and liver-specific (for example, miR-122a) miRNAs appear on the left-hand side.

In an alternative approach to analyzing the relative expression levels of all the predicted target genes of each miRNA within a particular tissue, we adapted the 'ranked ratio' (RR) described by Yu *et al*. [23]. They first ranked the expression levels of each gene across a series of tissues. For each tissue the ranked genes were divided into two halves, one with high and one with low ranks. The RR values were then calculated by dividing the number of targeted genes in the 'low' ranked group by the 'high' ranked group. Instead of considering a range of tissues we ranked the targeted genes within a single expression dataset and for each miRNA calculated an RR value by dividing the number of predicted target genes with expression levels below the median absolute expression value by the number of predicted target genes above this value (comparison with other methods suggested that this was more effective than dividing genes into upper and lower halves - see below). This RR value is, therefore, an indicator of the distribution of a miRNA's target genes within a single mRNA population. A high RR indicates low expression in a greater proportion of target genes and is, therefore, indicative of miRNA expression in that tissue. The RR values for all miRNAs were calculated for all eight tissues and the ranked RR values for brain and liver are shown in Figure 2 (for all other tissues analyzed, see Additional data file 2). As expected, known tissue-specific miRNAs have high RR values in their cognate tissue.

### Mean absolute expression

**Figure 3:**
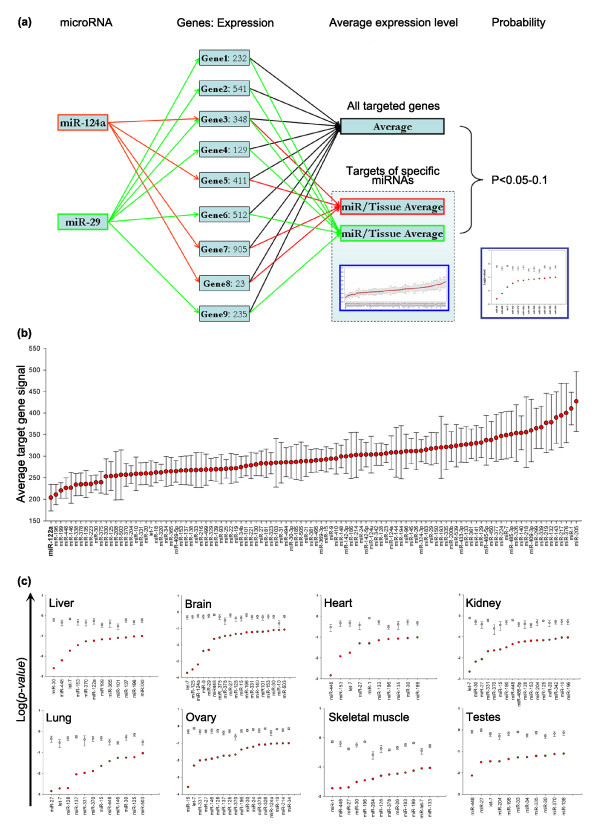
Average mRNA expression of the predicted target genes for each miRNA. **(a) **Schematic diagram illustrating how the average expression levels of individual miRNA predicted target gene sets are calculated and then compared with that of all predicted target genes. In this example miR-124a and miR-29 are shown to map to different, but overlapping, subsets of target genes. The average expression values of all predicted target genes and the miR-124a and miR-29 predicted targets are calculated. The probability that the expression levels of the genes predicted to be targeted by miR-124a and miR-29 in this tissue are drawn at random from the expression levels of all predicted target genes is calculated. **(b) **Ranked mean expression values (y-axis) of all predicted target genes for each miRNA (x-axis) with target genes expressed in liver are depicted as red circles (± standard error). These include the predicted mRNA targets of the known liver-specific miR-122 (extreme left). Several miRNAs have higher than expected target gene expression, for example, miR-1 and miR-205 (extreme right). **(c) **Red circles indicate the probability (log_10_, y-axis) that the set of target gene expression levels for each miRNA (x-axis) is drawn at random from the whole population of expressed target genes for all miRNAs (*t*-test, *p *< 0.1). For each miRNA the mean probability (± standard error) derived from five random sets of predicted target genes is plotted in grey.

We next investigated whether an approach involving absolute target gene expression could be used to detect miRNA signatures. This could potentially identify miRNAs missed above, but runs the risk of being unduly influenced by single genes with a large change in expression. The technique is outlined in Figure 3a. The miRNAs were ordered by the mean expression value of their predicted target mRNAs, as shown for liver in Figure 3b. Of all the miRNAs in the liver, the lowest mean target gene expression value was that of miR-122a, a well characterized liver-specific miRNA [28]. To determine the likelihood that this observed reduction in mRNA expression is due to the selection of mRNAs with specific miRNA targets, we calculated the probability (*t*-test) that these samples are drawn at random from amongst all those genes expressed in the tissue and that contain a predicted miRNA target sequence. The resulting probabilities for all tissues are plotted in Figure 3c and those miRNAs with low target gene expression include many known tissue-specific examples, such as miR-124a in brain (*p *= 6.2 × 10^-4^) and miR-1 in skeletal muscle (*p *= 1.9 × 10^-2^).

**Figure 4:**
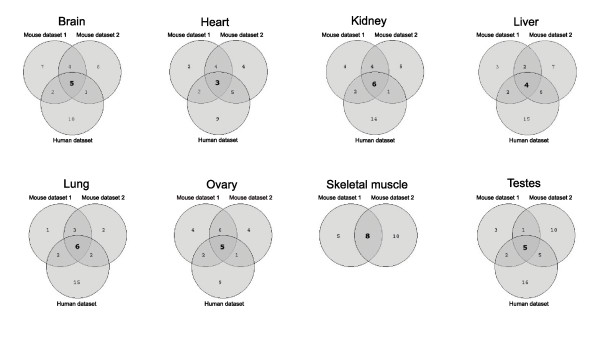
Correlation between miRNA signatures detected in two mouse and one human gene expression datasets. Results from eight tissues are presented (no suitable human skeletal muscle expression data were available) in separate Venn diagrams. Each circle in the Venn diagrams indicates the number of miRNAs with significantly low target gene expression in two mouse (top) and one human (bottom) mRNA expression datasets. The number of miRNAs common between all datasets in each tissue is indicated in bold.

To test the reliability of this approach and the robustness of available microarray expression data, it was applied to an independent mouse expression dataset generated in several different laboratories (see Materials and methods). The sets of miRNAs predicted from the two datasets were very similar for all tissues and the extent of overlap is depicted in Figure 4. Mammalian miRNAs and their target sites are highly conserved; indeed, sequence conservation is a requirement of the TargetScan predictions [4]. Accordingly, miRNA expression is conserved between species [29], at least for organisms with similar physiology [30] and miRNAs may have a role in reducing cross-species variation in mRNA expression [31]. Analysis of mRNA expression profiles from human tissues (Additional data file 3) revealed that approximately one-third of the human miRNAs with low target gene expression corresponded to those predicted in equivalent murine tissues (30.6% and 35.5% for mouse datasets 1 and 2, respectively). For example, of 18 human miRNAs predicted in brain, 7 were common with mouse dataset 1 (Figure 4), rather than the lower number (approximately 2) expected if the groups of miRNAs were independent (the observed numbers were similarly high for all other tissues and the second mouse dataset). This provides further evidence for conserved miRNA expression and independent validation of the prediction method.

### Comparison of miRNA signature detection methods

**Figure 5:**
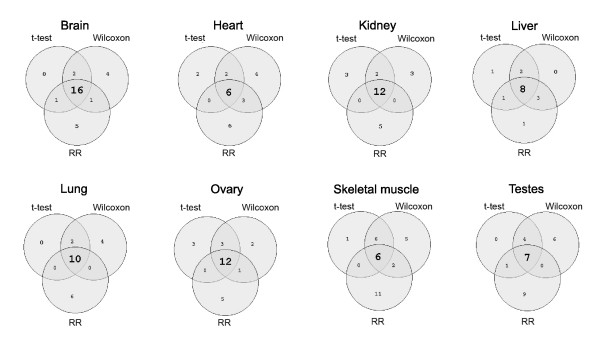
Overlap between three methods of detecting miRNA signatures on mRNA expression profiles. Results from eight tissues are presented in separate Venn diagrams. Each circle in the Venn diagrams indicates the number of miRNAs with significantly low target gene expression as predicted by the *t*-test, Wilcoxon or RR methods. For all tissues there was significant overlap amongst predicted miRNAs, with the Wilcoxon rank sum test and absolute expression *t*-test in strongest agreement. The numbers of miRNAs predicted by all three methods are indicated in bold.

We next compared the results of the three methods, Wilcoxon rank sum test, RR and absolute expression *t*-test, using a 10% significance level and an equivalent number of miRNAs from the RR method. For all tissues there was significant overlap amongst predicted miRNAs (Figure 5), with the Wilcoxon rank sum test and absolute expression *t*-test in strongest agreement. To evaluate how well the miRNA signature detected in target gene expression predicts actual miRNA expression, we compared the tissue distribution of miRNAs predicted by at least two of the methods with that derived from experimental evidence (cloning and Northern blots). Table 1 illustrates the accordance between the tissues in which miRNA activity (upon target genes predicted by TargetScan) is computationally predicted and those for which there is experimental evidence of miRNA presence (particularly when more recently characterized miRNAs are excluded). This is supported by positive Matthews correlation coefficients (MCC) [32] for all tissues, ranging from 0.2-0.5 (average value 0.34; Additional data file 4).

**Table 1:** Correlation between predicted and previously characterized tissue-specific miRNA expression

miRNAs	Brain	Heart	Kidney	Liver	Lung	Ovary	SM	Testes
let-7	*1,2,3	*1,2,3,4	*1,3,4,6	*2	*1,8	*	*1	*3,9
miR-1/206	2	*1,2,4,5	1	*2	1		1,5	
miR-10			*4,6,7			*7		
miR-101	*2		4	*1			*	9
miR-122a	3			*1,2,3				9
miR-124a	*1,2							
miR-125	*1,2	*1,3,4	*1,4	1	*1,3	*		
miR-128	*1,2		*		*1	*	*	
miR-133	2,3	*1,2	1				*1,2	
miR-136	2							*
miR-137	*1,2			*	*	*		
miR-140	*		4					
miR-144		2		*				
miR-146	3	2,4	4		*	*		
miR-15	*2,3	1,2,3	*4,6		*1,3	*		*3,9
miR-150			4			*		
miR-153	*1,2	*	*	*				
miR-190	1		*7					
miR-193			1,7,4		1		*	
miR-196	*	*	*1,4		3	*7	*	*
miR-199		4	4	1			*	
miR-204							*	*
miR-24	2,3	*2	1,4,6		*1,8	*		
miR-26	1,2,3	1,4	1,4,6	1,2	1		* 1	
miR-27	*2,3	*2	*4,6		*1,8	*	*	*9
miR-29	*1,2	2,4	*4,6		1			
miR-30	*1,2,3	*2,4	*1,4,6	*1,2	*1	*	*1	*9
miR-31	*							
miR-326		*				*		
miR-33			4				*	*
miR-330				*				
miR-331	* 3		*	*	*3	*		
miR-335								*
miR-34	3					*		*9
miR-365	3			*				
miR-370			*	*	*	*		*
miR-375	*							
miR-378	*			*			*	
miR-448	*	*	*	*	*		*	*
miR-503	*3	3	3		3			3
miR-9	*1,2							

All miRNAs for which expression of predicted target genes (according to TargetScan) was lower than expected in ≥ 1 tissue (*p *< 0.1) are listed and the tissues marked with an asterisk. Tissues in which miRNA expression has been experimentally characterized are indicated by the number of the appropriate reference: 1, Sempere *et al*. [29]; 2, Lagos-Quintana *et al*. [28]; 3, Gu J *et al*. [47]; 4, Naraba and Iwai [48]; 5, Zhao Y *et al*. [49]; 6, Lagos-Quintana *et al*. [50]; 7, Lagos-Quintana *et al*. [51]; 8, Hayashita *et al*. [52]; 9, Yu *et al*. [53]). Cells with both a reference number and asterisk indicate the overlap between our predicted expression pattern and the experimental data. SM, skeletal muscle.

### Correlation of miRNA signatures with miRNA expression levels

**Figure 6:**
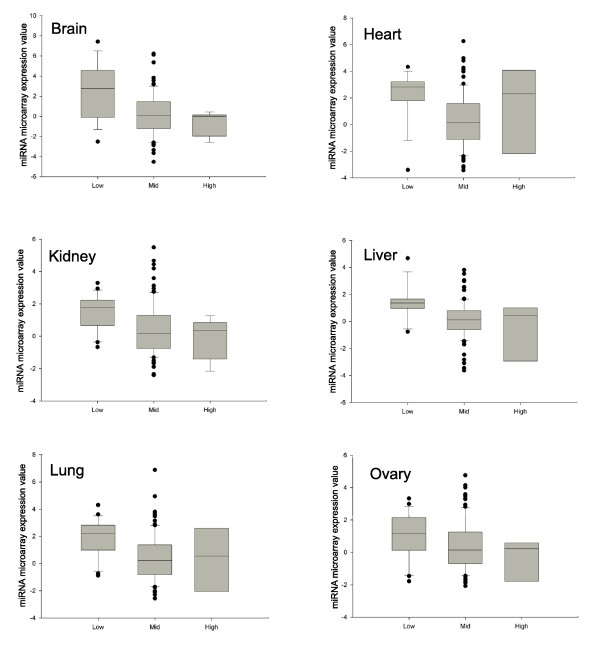
Correlation between miRNAs with predicted effects on mRNA expression and miRNA expression levels detected by miRNA microarrays. miRNAs were divided into those with significantly lower than expected target mRNA expression (labeled 'low'), those with no detectable effect on their target expression (labeled 'mid') and those with significantly high target expression (labeled 'high'). The boxplots show the expression values (y-axis), determined by Thomson *et al*. [33], of the miRNAs in each group (x-axis). The expression of miRNAs with low target gene expression is significantly higher (*t*-test, *p *< 0.05) than that of those with mid or high target expression (in all tissues except heart). Thomson *et al*. [33] labeled the microRNAs from each tissue with Cy3 and used a reference oligonucleotide set corresponding to all mature microRNAs, labeled with Cy5 (red channel) in all hybridizations. This reference set provided an internal hybridization control for every probe on the array. The miRNA microarray expression values used in our analyses are median centered normalized log ratio Cy3/Cy5 values.

miRNA microarrays are now available that provide a global indication of miRNA expression within a tissue. We therefore compared our predictions of miRNAs that alter mRNA expression with the actual expression of the miRNAs themselves, as determined by miRNA microarrays [33]. For all tissues the expression levels of miRNAs with low target gene expression, determined by the absolute expression method (10% significance level), were significantly lower (*t*-test, *p *< 0.05) than those miRNAs having no detectable effect on their target genes (Figure 6). This provides further confirmation that the miRNA signatures we have detected are a consequence of miRNA expression in the cognate tissue. In addition to miRNAs with low target gene expression, we detected a set of miRNAs whose target genes were expressed at significantly higher levels than the background set (Figure 3b). The expression of these miRNAs, as determined by microarrays, was not significantly different from those with no effect on mRNA expression.

**Figure 7:**
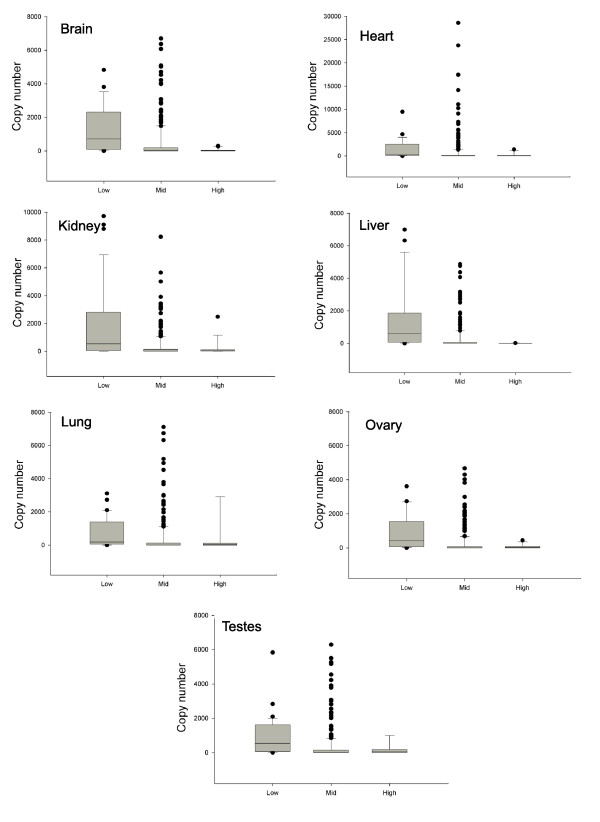
Correlation between miRNAs with predicted effects on mRNA expression and miRNA expression levels detected by RT-PCR. miRNAs were divided into groups according to predicted target gene expression in human tissues, as described previously. The expression, as determined by RT-PCR [34] (y-axis), of the miRNAs in these groups (x-axis) is depicted by boxplots that illustrate the significantly higher expression (*t*-test, *p *ranging from 0.0014 in kidney to 0.0385 in brain) of miRNAs with low target gene expression relative to those with mid or high target expression (in all tissues except heart). Where necessary to present the median and interquartile ranges effectively, up to two outliers were omitted.

Recently, comprehensive miRNA expression data for human tissues determined by reverse transcription PCR (RT-PCR) have become available [34]. This revealed an even clearer relationship between human miRNA copy number and level of predicted target gene expression (Figure 7). In an attempt to demonstrate the similarity between human and mouse miRNAs, the human orthologs of miRNAs predicted from analysis of murine data to have 'low' or 'mid' predicted target gene expression were selected. Surprisingly, significant differences were detected between the copy numbers in human tissues of these two groups of miRNAs, which had been selected based upon murine target gene expression (Additional data file 5). This is testament to the degree of conservation of miRNAs and their target genes between mice and humans and the accuracy of the RT-PCR measurements.

Some of those miRNAs with a significant effect on mRNA expression were highly expressed (for example, miR-122a, miR-124a, miR-125) and their observed lowering of mRNA levels could reasonably be attributed to a weak mRNA degradative activity secondary to their principal action directed at translation. However, other miRNAs that significantly affected target gene expression were not highly expressed, perhaps indicating a greater efficiency in mRNA degradation for these particular miRNAs. Therefore, the extent to which specific miRNAs cause mRNA degradation might be influencing our ability to detect their presence. We reasoned that the difference between miRNAs would be most marked between those highly expressed but having no detectable affect on mRNA expression and those expressed at a low level but with a significant impact on target mRNA expression. Other than extensive complementarity [9], the features of the miRNA-target interaction required for miRNAs to direct mRNA cleavage are unclear, although a number of features of site context, including position, local AU content and pairing with miRNA 3' residues have been shown to increase site efficacy [2]. In a preliminary attempt to characterize the distinguishing properties of the potential classes of miRNAs described above, we analyzed the lengths of contiguous complementarity between miRNAs and predicted sites, but there were no significant differences.

### Perturbation of miRNA expression affects cognate mRNA expression

**Figure 8:**
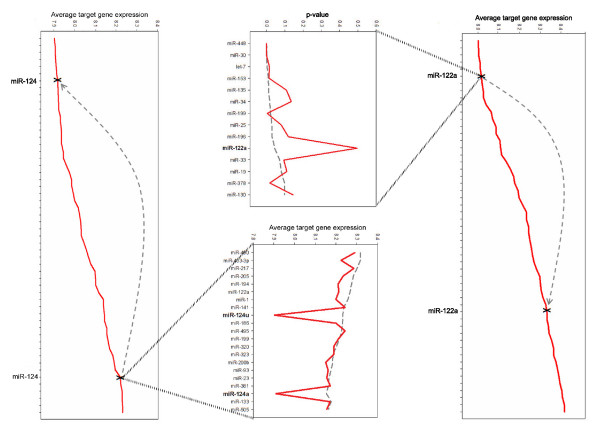
Detection of altered miRNA signatures following manipulation of miRNA expression. In the top and bottom panels miRNAs (x-axis) are ranked in order of average target gene expression (y-axis) with, for clarity, only miR-122a and miR124 labeled, respectively. Following inhibition of miR-122 by an antagomir [13] the position of the miR-122 average target gene expression in liver moved from the left to right of those for all ranked miRNAs (upper panel). This was reflected in a reduced probability (y-axis) that miR-122a predicted target genes were under-expressed, whilst all other miRNA effects remained relatively unaltered by the miR-122a antagomir (red versus dashed grey line in center left panel). Conversely, overexpression of miR-124 for 24 hours in the HepG2 cell line [12] caused miR-124 target gene expression to change from significantly highly over-expressed to significantly under-expressed (bottom panel). The selective drop in expression of miR-124 target gene expression is shown in the center right panel (this is apparent for the miR-122a and miR-122u isoforms present in the version of TargetScan used for this analysis). Whilst the data in this Figure are from analysis of mean absolute expression, the alternative methods produced similar results (data not shown).

In order to validate the miRNA signatures observed in mRNA profiles of normal tissues, we applied our approach to several publicly available gene expression datasets measured following an experimental perturbation of miRNA expression that resulted in either a decrease [13] or increase [12] in the activity of a specific miRNA. In each case the expected response was observed in target gene expression. Krutzfeldt *et al*. [13]demonstrated that intravenous injection of chemically modified oligonucleotides, or 'antagomirs', complementary to miRNAs could specifically reduce the endogenous levels of the corresponding miRNA in mice. When we analyzed gene expression data from liver in which miR-122a had been silenced by use of an antagomir [13] the list of miRNAs with an influence on mRNA levels was very similar but miR-122a no longer had a detectable effect (Figure 8). This conclusively demonstrates that the signature of miR-122a on target gene expression that we observe is due to the physical presence of miR-122a rather than any evolutionary pressure on the expression of target genes co-expressed with their cognate miRNA.

Wang and Wang [12] transfected the HepG2 cell line with RNA duplexes that mimicked the miR-124 precursor. They demonstrated the effect of miR-124 over-expression by measuring mRNA expression profiles and showing that predicted targets of miR-124 were over-represented amongst the mRNAs down-regulated following this treatment, compared to a negative control RNA duplex. As expected, analysis of miRNA signatures in these expression datasets revealed miR-124 activity in cells in which it was overexpressed but not in controls (Figure 8).

### Characteristics of predicted target gene groups

We could not find any increase in target complementarity for miRNAs with a significant effect on mRNA levels. However, we noticed that those miRNAs with low overall target gene expression in a specific tissue generally had a greater number of target genes expressed in that tissue. This was true across tissues and supports the observations of Farh *et al*. [17], who demonstrated that tissue-specific miRNA target genes are generally expressed in the cognate tissue, but at lower levels than other tissues.

In certain tissues some miRNA predicted target gene sets were expressed at significantly higher levels (10% probability) than those of other miRNAs. We observed that these often involved a highly expressed, tissue-specific miRNA in tissues in which the miRNA was not normally expressed, for example, miR-1 in liver (Figure 2). However, this phenomenon does not appear to be indicative of reduced suppression of miRNA levels due to low miRNA expression in these tissues (Figures 6 and 7).

The targeting of mRNAs by miRNAs has been associated with 3' UTR GC content and length [16,35]. However, we found no consistent differences between the 3' UTR GC content of predicted target genes of miRNAs with significantly low target gene expression ('low' genes) and those of background genes. However, for all tissues, the predicted target genes of miRNAs with significantly high target gene expression ('high' genes) had significantly lower GC content (Additional data file 6). The 3' UTR length was significantly longer than background for 'low' genes, but 'high' genes were even longer. The biological significance of these observations is unclear. One might have expected genes with lower expression to be more AU rich because miRNAs have been implicated in degradation of mRNAs containing AU-rich elements [35]. The longer 3' UTRs of 'low' genes would be predicted to have more miRNA target sites and, therefore, be subject to more degradation, but this is inconsistent with the even longer 3' UTRs observed for 'high' genes.

### Use of alternative target prediction methods

The correlation demonstrated between expression of certain miRNAs and that of their predicted target genes indicates that at least some of the interactions suggested by TargetScan are valid. Random assignment of miRNA target genes rather than use of TargetScan predictions resulted in fewer miRNAs with low target gene expression and significantly less overlap between those miRNAs with low target gene expression in replicate expression datasets (*p *< 0.00001).

We next investigated whether we could detect variations in expression of the targets predicted by alternative algorithms. Many of the highly expressed, known tissue-specific miRNAs identified above (Table 1) were not observed when the equivalent analyses were performed with target gene sets predicted by miRanda [36,37] or RNAhybrid [38,39]. For example, in brain the TargetScan-based analyses predicted 14 of 24 known miRNAs whilst miRanda and RNAhybrid predicted 4 and 5, respectively. In Liver, TargetScan, miRanda and RNAhybrid predicted five, one and one, respectively, out of eight, and in skeletal muscle five, three and one out of five. The predicted targets of miR-122a were not lowly expressed in liver and, therefore, no effect could be observed following treatment with antagomir. Likewise, the expression levels of the predicted targets of miR-124 were not significantly lower following its overexpression. However, the miRNAs with low predicted target gene expression in a particular tissue were expressed at higher levels than others in that tissue (Additional data files 7 and 8), albeit less markedly than those miRNAs derived from equivalent analyses with TargetScan predictions (Additional data file 5). This suggests that these prediction methods do detect real miRNA targets, but that there may be more false predictions and/or that the target genes detected by these criteria are less susceptible to miRNA-mediated reduction in mRNA levels. The miRNAs detected in the analyses based upon miRanda or RNAhybrid target predictions are listed in Additional data file 9.

It has been suggested that target mRNAs can be repressed by miRNA binding sites in the 5' UTR [40]. Analysis of all mouse 5' UTRs for target sites using RNAhybrid yielded fewer predictions than in 3' UTRs and there was no obvious link between miRNAs with low target gene expression and known, highly expressed, tissue-specific miRNAs such as miR-1 in heart or miR-122a in liver. However, there is evidence from the miRNA expression data of Liang *et al*. [34] that those miRNAs with low predicted target gene expression are more highly expressed (Additional data file 10). This suggests that functional interactions are occurring between miRNAs and 5' UTRs *in vivo*.

## Conclusion

It is perhaps surprising that given the wide range of regulatory mechanisms acting on gene expression, we are able to detect the effect of miRNAs on their diverse collection of target genes. The effectiveness of three analytical approaches, including the use of absolute expression values that might be unevenly skewed by single extreme values, illustrates the extent of the effect and the correlation between independent datasets is remarkable. These findings provide further evidence that miRNAs play an important role in regulating mRNA expression and can affect the mRNA levels of multiple genes. Although the effects on mRNA expression profiles are highlighted following artificial manipulation of miRNA levels [12,13], these are real *in vivo *effects because they are observed in data derived from endogenous situations. One limitation of the TargetScan predictions that we used, is that they cannot discriminate between miRNA family members with the same seed sequence (this has been addressed in release 4.0).

The mechanism by which miRNAs are causing degradation of their cognate mRNAs is unclear [8,41] and may be a side-effect of the translation inhibition process and be related to high miRNA expression levels. In rare cases miRNAs can direct slicer-mediated cleavage if there is extensive complementarity with the target site, as reported for miR-196 and HOXB8 mRNA [9]. This is not the case for the miRNA-target interactions reported here, but they may be inducing deadenylation and subsequent cleavage [42]. The ability of specific miRNAs to reduce the level of certain target mRNAs very efficiently might explain why they have a detectable effect upon gene expression in tissues in which their expression has been shown to be very low, for example, miR-196 in brain. This raises the possibility that predicted miR-196 target sites included in this analysis may have distinctive features. An updated release of TargetScan (4.0) includes scores for features that contribute to predicted target site efficacy, which are summarized in a 'context score' [2]. The context scores for the predicted targets of miR196 expressed in brain are not significantly higher than those of other miRNAs. However, there are several predicted target genes, including *HOXB7 *and *HOXA5*, with high context scores - efficient degradation of these targets could contribute to a detectable signal from low levels of miR-196. Analyses of miRNA signatures in gene expression profiles will help to pinpoint those miRNAs acting at the mRNA level and determine to what extent this results from high miRNA expression or reflects specific mechanisms related to certain miRNAs.

It is difficult to validate miRNA target predictions from effects on translation due to the technical difficulties of genome-wide quantitative protein profiling. Analysis of the effects of miRNAs on target mRNA expression provides a potential alternative. The target predictions we employed [27,37,39] were sufficiently accurate to enable detection of miRNA signatures within gene expression profiles. The ability to detect these miRNA signatures also attests to the sensitivity and reproducibility of current microarray technology. The strength of signal detected could be used to assess the effectiveness of different target prediction algorithms. The lack of significant effects in previous studies [16] might be because the target gene predictions employed were less effective at predicting miRNA-target interactions that result in mRNA degradation.

A unique advantage of this approach is that it is possible to infer miRNA activity from a single gene expression profile rather than having to first rank the expression of each gene across a series of experiments [11,16,17]. This will facilitate the analysis of individual datasets of interest from the vast repository of gene expression data that are steadily accumulating [20,22]. It is applicable to data from any sufficiently sensitive and comprehensive platform and to gene expression profiles generated from biological sources too scarce to permit direct miRNA detection.

Our analysis of a limited number of gene expression profiles has demonstrated the major impact of miRNAs upon mRNA expression. The programs are amenable to provision of an online service that would enable individual researchers to gain an indication of miRNA activity in their tissue/cells of interest. This is a convenient approach to gain additional biological insight from gene expression profiles.

## Materials and methods

Stand Alone Java and R programs [43], in conjugation with MS Access and MS Excel, were used for the following analyses.

### Gene expression data

Mouse and human mRNA expression datasets that covered a range of tissues were downloaded from GEO [20,21,44]. In addition, a second mouse dataset covering the same tissues was compiled from data submitted by different laboratories. The tissues and GEO sample and platform accession numbers are listed in Additional data file 3. Evidence for tissue-specific miRNA expression supported by cloning, Northern hybridization or expressed sequence tag mapping was determined by literature review.

### Calculation of miRNA target gene expression

Only single gene-specific Affymetrix probesets (suffix_at) were considered and only those expressed in each dataset analyzed (designated by a 'present' call if available, or alternatively a signal strength greater than the median value). Probeset IDs were converted to their cognate gene symbols, using the Biomart online suite [45]. Lists of miRNA families and their predicted target genes published by Lewis *et al*. [4] were downloaded from the TargetScan website (version 3.1) [27]. For mouse (Mouse Genome 430 2.0 genechip), 15,682 probesets were mapped to gene symbols and of these, between 3,120 and 3,792 were both predicted miRNA targets and expressed in the tissues analyzed (for human, 17,325 probesets and 4,135-5,906 expressed targets were analyzed). Additional miRNA target genes predicted by miRanda [36,37] were downloaded from the miRBase website [46]. Sites with scores and conservation values (P_org) greater than average were selected for analysis. The RNAhybrid algorithm [39] was run with the following parameters: *p *< 0.05, energy < -10 kcal/mol and with enforced binding to bases 2-7.

For each mRNA expression dataset, lists of the expressed genes predicted to be targeted by each miRNA and their respective expression levels were compiled (the average value was calculated for genes represented by > 1 probeset). Only miRNAs with > 50 predicted targets were considered further. These were sorted according to the average expression values (absolute or logarithmically transformed according to the primary microarray normalization algorithm used to analyze the data) of their predicted target genes and when necessary divided into upper and lower halves.

### Statistical analysis

Three different analytical methods were used to compare the expression of the predicted targets of a specific miRNA in a particular tissue, with the set of expression values of all the genes predicted to be targeted by miRNAs in the same tissue. To analyze whether there was significant difference between the medians of the ranked gene expression values of the two sets, the nonparametric one-sided Wilcoxon rank sum test was employed. To test the hypothesis that the genes in each set are from the same population, we used a one-tailed Student's *t*-test (considering unequal variance and using log-normalized data). For every miRNA with *p *< 0.1, we considered that the expression of all the genes predicted to be targeted by that miRNA in that tissue was significantly more or less than the average expression of all the predicted target genes.

To calculate an RR value, predicted target genes were ordered by expression value and then divided into two groups by either the median gene rank or absolute expression value. For each miRNA, the number of genes targeted in the lower group was divided by the number in the higher to give an RR value.

MCCs [32] were calculated as a measure of performance.

### Calculation of GC content and length of predicted target mRNA 3' UTRs

Mouse 3' UTR data were downloaded from Biomart [45]. The GC content and the lengths of the UTRs were then calculated for the nonredundant sets of genes predicted to be targeted by groups of miRNAs of interest.

### Analysis of miRNA-target site complementarity

For sets of miRNAs of interest, the number of predicted targeted genes having greater than or equal to *x *contiguous complementary nucleotides after the 6 bp seed was noted (where *x *ranged from 0-8, 0 being the seed itself and 8 the maximum complementarity observed after the seed region). The number of predicted target genes was calculated with and without consideration of multiple miRNA family members.

## Abbreviations

GEO, Gene Expression Omnibus; MCC, Matthews correlation coefficients; miRNA, microRNA; RR, ranked ratio; RT-PCR, reverse transcription PCR; UTR, untranslated region.

## Authors' contributions

DACS conceived the study. AA collected the data and performed the data analyses. DACS and AA interpreted the results. DACS drafted the manuscript with assistance from AA. Both authors read and approved the final manuscript.

## Additional data files

The following additional data are available with the online version of this paper. Additional data file 1 is a figure showing the number of target genes predicted by TargetScan for each miRNA and expressed in each mouse tissue. Additional data file 2 is a figure displaying the RR values for all miRNAs in heart, kidney, lung, ovary, skeletal muscle and testes. Additional data file 3 is a table listing the sources of gene expression data from the NCBI GEO website. Additional data file 4 is a table listing the MCCs calculated for each tissue. Additional data file 5 is a series of box plots showing the correlation between mouse miRNAs with lowly expressed mouse target genes predicted by TargetScan and expression levels of orthologous human miRNAs detected in cognate tissues by RT-PCR. Additional data file 6 is a figure showing the GC content and lengths of the 3' UTRs of miRNA predicted target mRNAs in a range of tissues. Additional data file 7 is a series of box plots showing the correlation between miRNAs with lowly expressed target genes predicted by miRanda and miRNA expression levels detected by RT-PCR. Additional data file 8 is a series of box plots showing the correlation between miRNAs with lowly expressed target genes predicted by RNAhybrid and miRNA expression levels detected by RT-PCR. Additional data file 9 is a table listing miRNAs with significantly low target gene expression determined by miRanda and RNAhybrid.

Additional data file 10 is a series of box plots showing that miRNAs with lowly expressed predicted target genes, as defined by putative 5' UTR sites, are expressed at higher levels than those with no effect on target gene expression.

## Supplementary Material

Additional data file 1 The number of predicted target genes (y-axis) for each miRNA (x-axis) is displayed with a different symbol for each tissue (grey circle, brain; green triangle, kidney; blue square, lung; green diamond, skeletal muscle; white circle, heart; red triangle, liver; purple square, ovary; yellow diamond, testes). miRNAs with less than 50 predicted target genes expressed in a specific tissue were not considered (see dashed line). Click here for file

Additional data file 2 Each panel displays the data from a single tissue. The miRNAs are arranged in ascending order according to RR value, shown on the left-hand y-axis and displayed as a red line. The higher values reflect lower target gene expression and are, therefore, indicative of miRNA activity. The number of predicted target genes is shown on the right-hand y-axis and the values for each miRNA indicated by a dashed line. Click here for file

Additional data file 3 Sources of gene expression data from the NCBI GEO website. Click here for file

Additional data file 4 The parameters were obtained from the predictions and evidence presented in Table 1. Click here for file

Additional data file 5 Box plots depict the copy numbers (y-axis) of groups of microRNAs (x-axis) whose mouse orthologs have low, mid or high predicted target gene expression (as defined in the text). Where necessary to present the median and interquartile ranges effectively, up to two outliers were omitted. Click here for file

Additional data file 6 The graphs on each row show data from a single tissue, with the first column depicting 3' UTR length and the second GC content. The predicted target genes are divided into three groups, low (red), medium (orange) and high (yellow). Those in the low group are targeted by miRNAs with overall significantly low target gene expression, those in the medium group are targeted by miRNAs with overall target gene expression within the expected range and those in the high group are targeted by miRNAs with overall high target gene expression. The average lengths in bases or %GC content (y-axis) of the 3' UTRs of each group of genes (x-axis) are shown with standard error bars. Click here for file

Additional data file 7 miRNAs were divided onto those with significantly lower than expected target mRNA expression (labeled 'low') and those with no detectable effect on their target expression (labeled 'mid'). The boxplots show the copy number (y-axis) of the miRNAs in each group (x-axis) and illustrate the significantly higher expression of miRNAs with low target gene expression. The scale of the y-axis (copy number) was chosen to facilitate visual comparison between groups and necessitated omission of up to three outliers from several graphs. Click here for file

Additional data file 8 miRNAs were divided into those with significantly lower than expected target mRNA expression (labeled 'low') and those with no detectable effect on the expression of their predicted targets (labeled 'mid'). The boxplots illustrate the significantly higher expression of miRNAs with low target gene expression. The scale of the y-axis (copy number) was chosen to facilitate visual comparison between groups and necessitated omission of up to three outliers from several graphs. Click here for file

Additional data file 9 miRNAs with significantly low target gene expression determined by miRanda and RNAhybrid. Click here for file

Additional data file 10 miRNAs were divided into groups according to gene expression of targets predicted from potential 5' UTR miRNA binding sites. For each tissue (brain, heart, kidney, liver, lung, ovary, skeletal muscle and testes) a box plot shows the miRNA copy number, as determined by RT-PCR [34] (y-axis) of the miRNAs in each group ('low' and 'mid', x-axis). Click here for file
