# Supplementary figures and images for: Individual mRNA expression profiles reveal the effects of specific microRNAs
Source: Genome Biol. 2008 May 16;9(5):R82. doi: 10.1186/gb-2008-9-5-r82 (PMC2441468; doi:10.1186/gb-2008-9-5-r82)

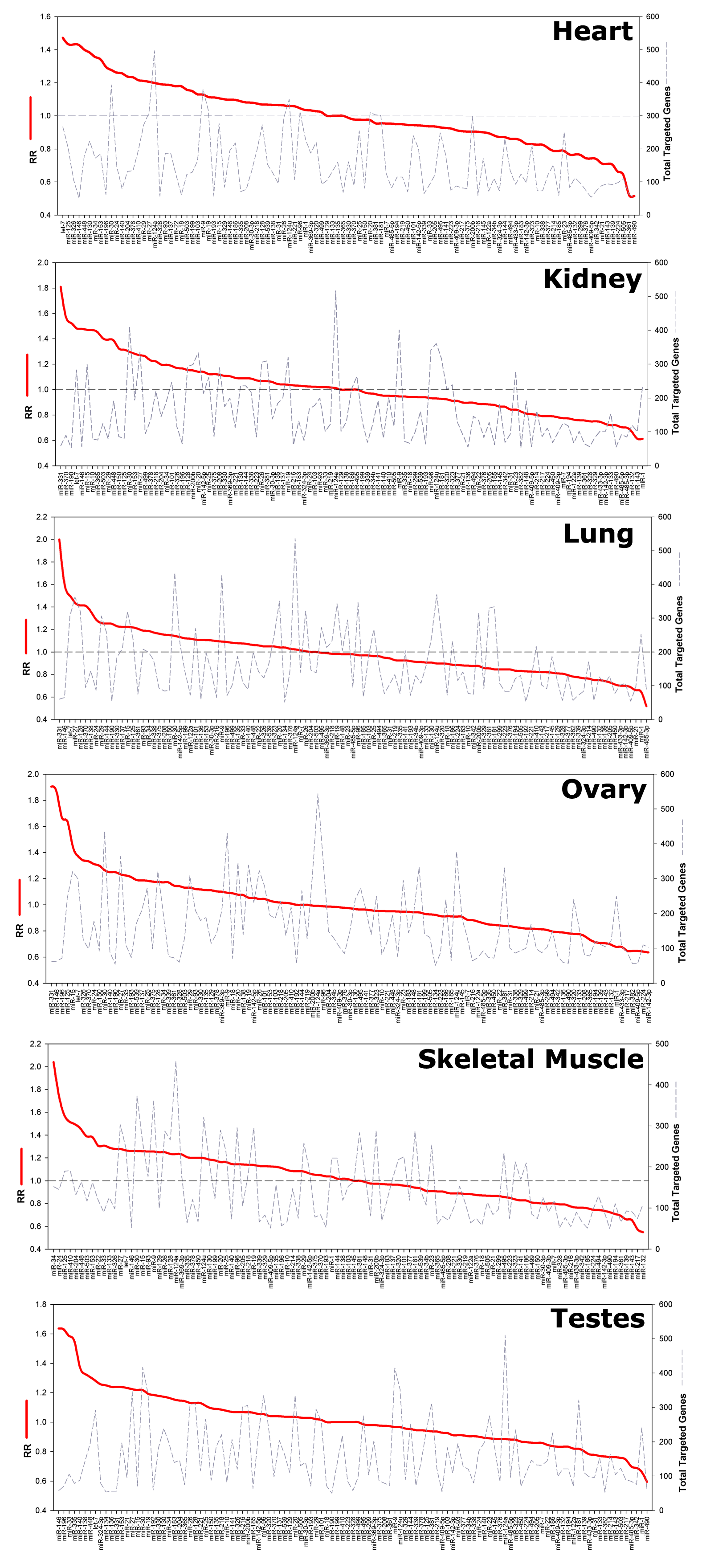

Supplement: Additional data file 2 — Each panel displays the data from a single tissue. The miRNAs are arranged in ascending order according to RR value, shown on the left-hand y-axis and displayed as a red line. The higher values reflect lower target gene expression and are, therefore, indicative of miRNA activity. The number of predicted target genes is shown on the right-hand y-axis and the values for each miRNA indicated by a dashed line. [file gb-2008-9-5-r82-S2.tiff]

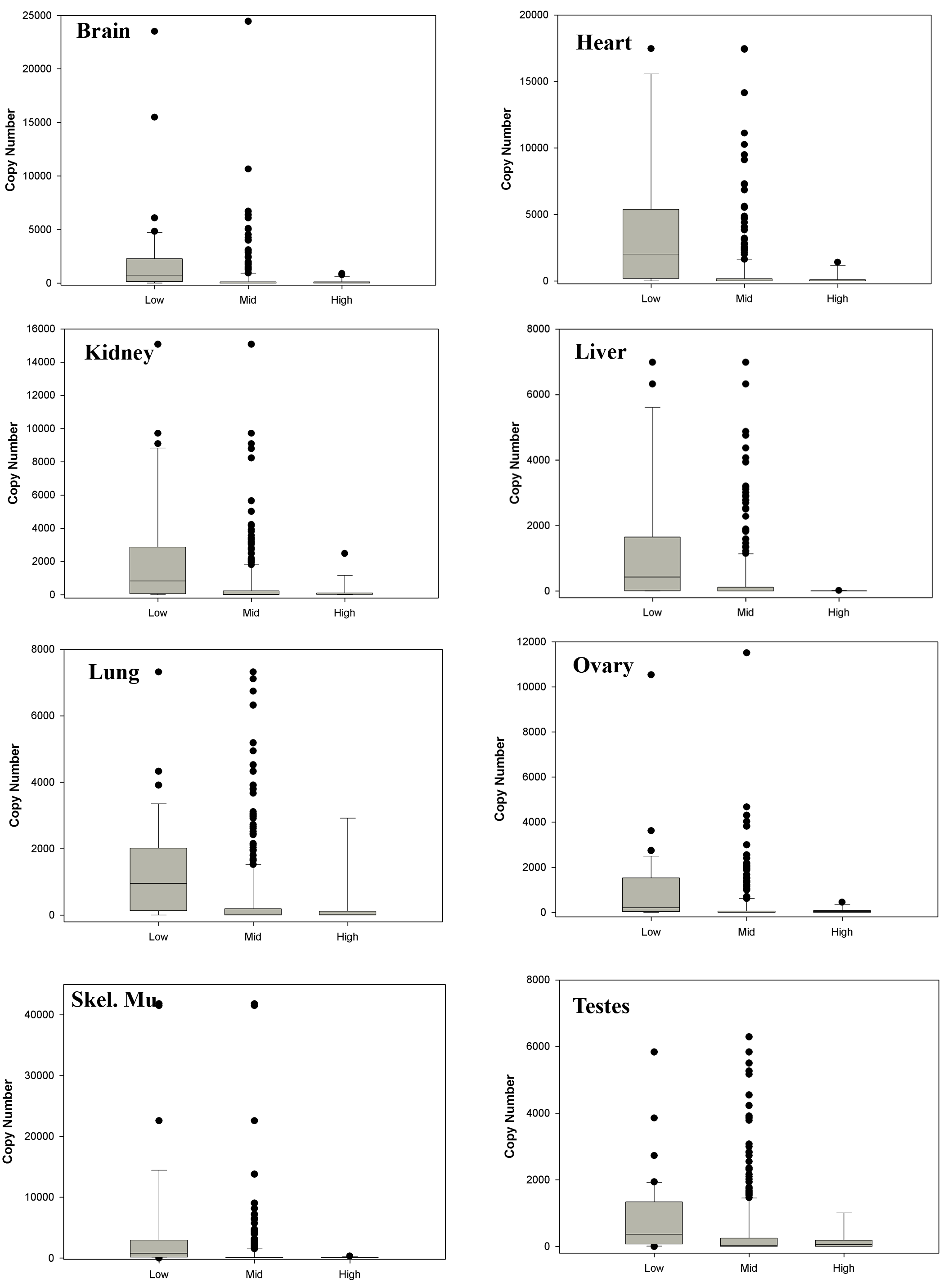

Supplement: Additional data file 5 — Box plots depict the copy numbers (y-axis) of groups of microRNAs (x-axis) whose mouse orthologs have low, mid or high predicted target gene expression (as defined in the text). Where necessary to present the median and interquartile ranges effectively, up to two outliers were omitted. [file gb-2008-9-5-r82-S5.tiff]

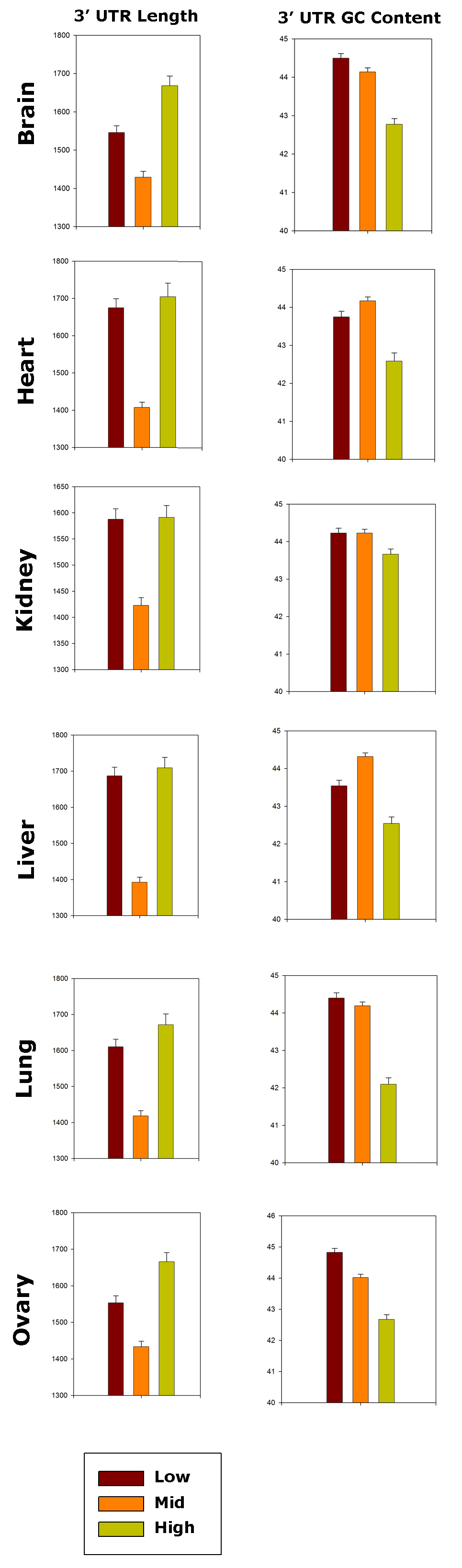

Supplement: Additional data file 6 — The graphs on each row show data from a single tissue, with the first column depicting 3' UTR length and the second GC content. The predicted target genes are divided into three groups, low (red), medium (orange) and high (yellow). Those in the low group are targeted by miRNAs with overall significantly low target gene expression, those in the medium group are targeted by miRNAs with overall target gene expression within the expected range and those in the high group are targeted by miRNAs with overall high target gene expression. The average lengths in bases or %GC content (y-axis) of the 3' UTRs of each group of genes (x-axis) are shown with standard error bars. [file gb-2008-9-5-r82-S6.png]

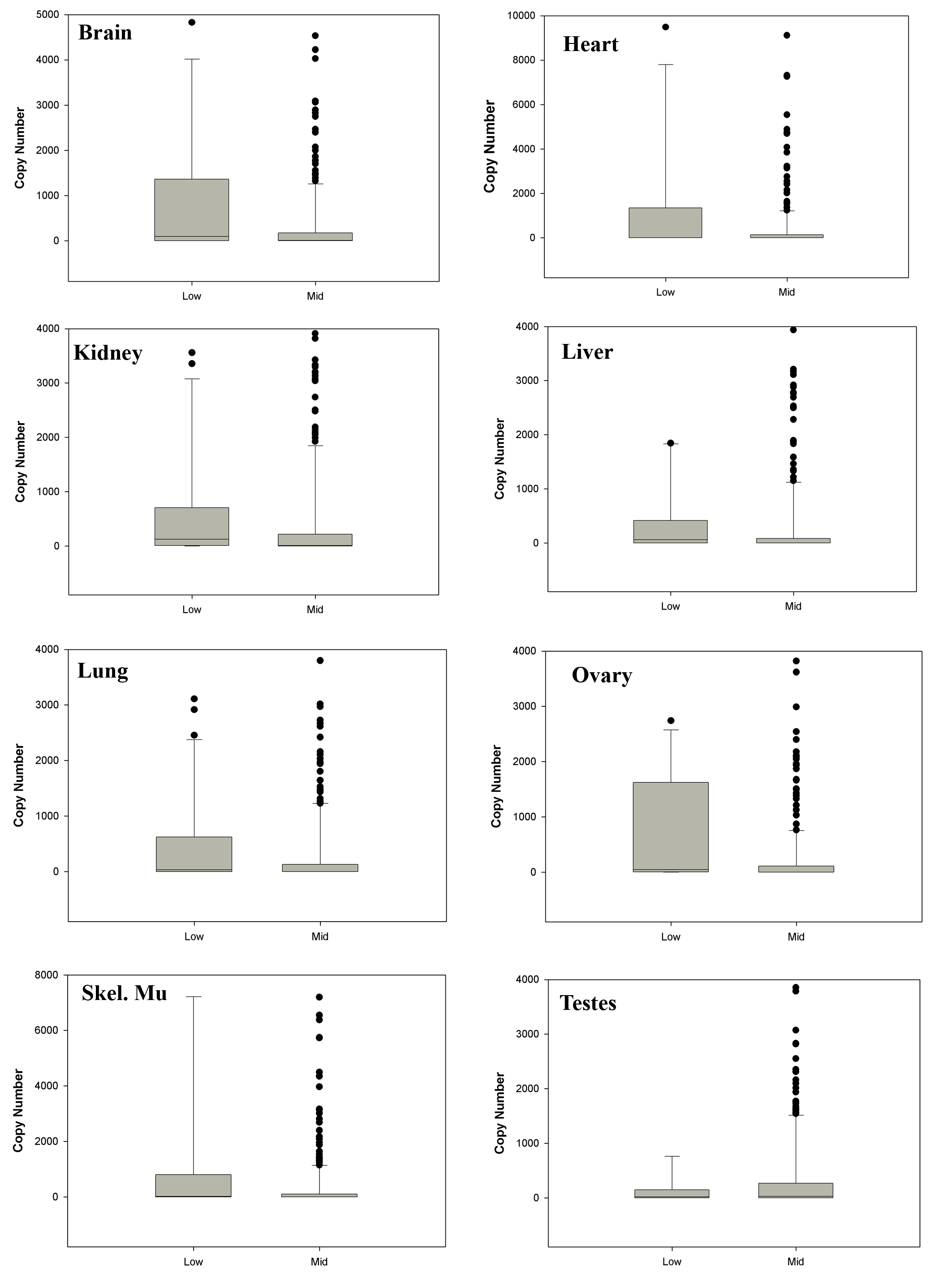

Supplement: Additional data file 7 — miRNAs were divided onto those with significantly lower than expected target mRNA expression (labeled 'low') and those with no detectable effect on their target expression (labeled 'mid'). The boxplots show the copy number (y-axis) of the miRNAs in each group (x-axis) and illustrate the significantly higher expression of miRNAs with low target gene expression. The scale of the y-axis (copy number) was chosen to facilitate visual comparison between groups and necessitated omission of up to three outliers from several graphs. [file gb-2008-9-5-r82-S7.tiff]

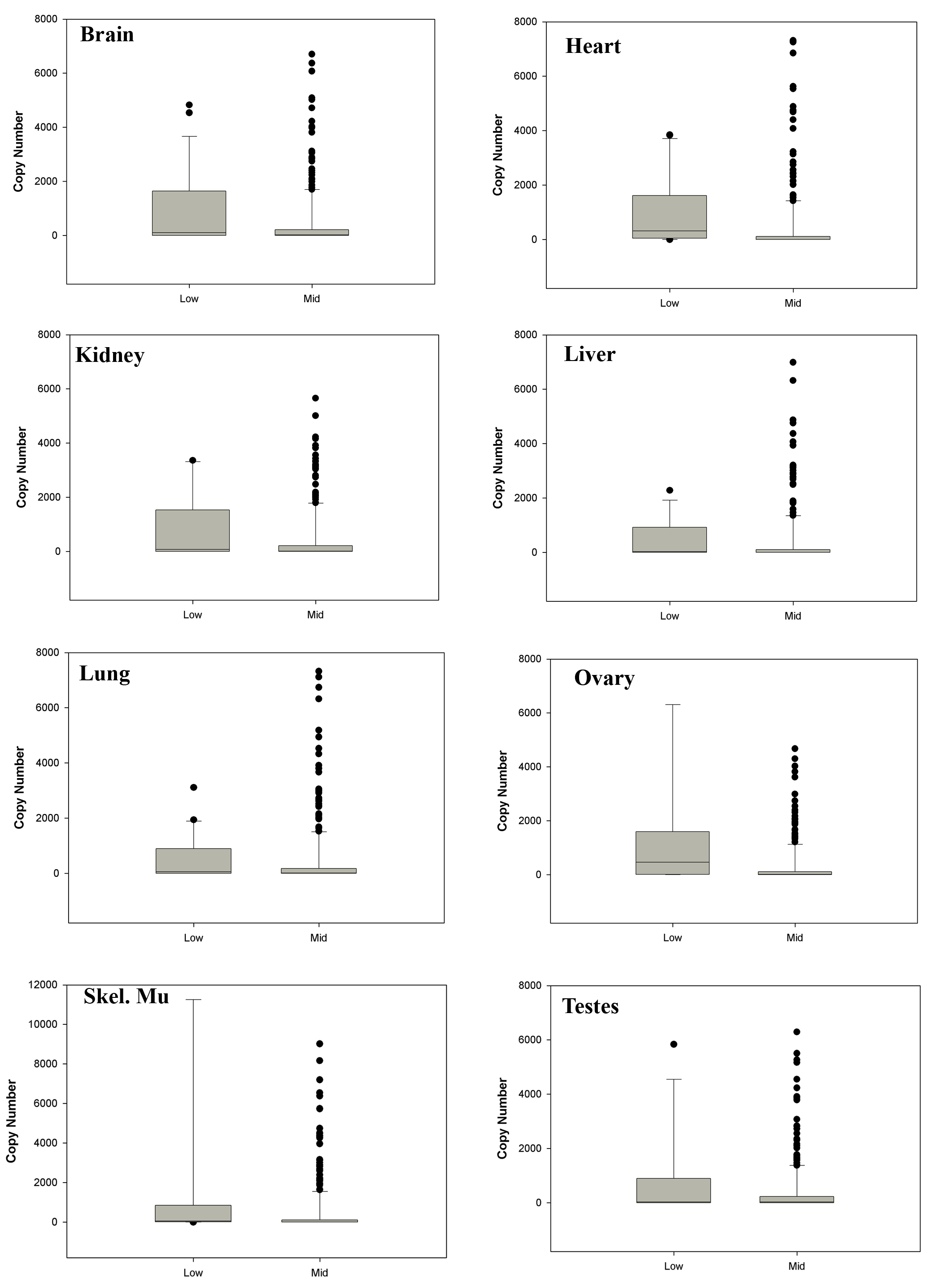

Supplement: Additional data file 8 — miRNAs were divided into those with significantly lower than expected target mRNA expression (labeled 'low') and those with no detectable effect on the expression of their predicted targets (labeled 'mid'). The boxplots illustrate the significantly higher expression of miRNAs with low target gene expression. The scale of the y-axis (copy number) was chosen to facilitate visual comparison between groups and necessitated omission of up to three outliers from several graphs. [file gb-2008-9-5-r82-S8.tiff]

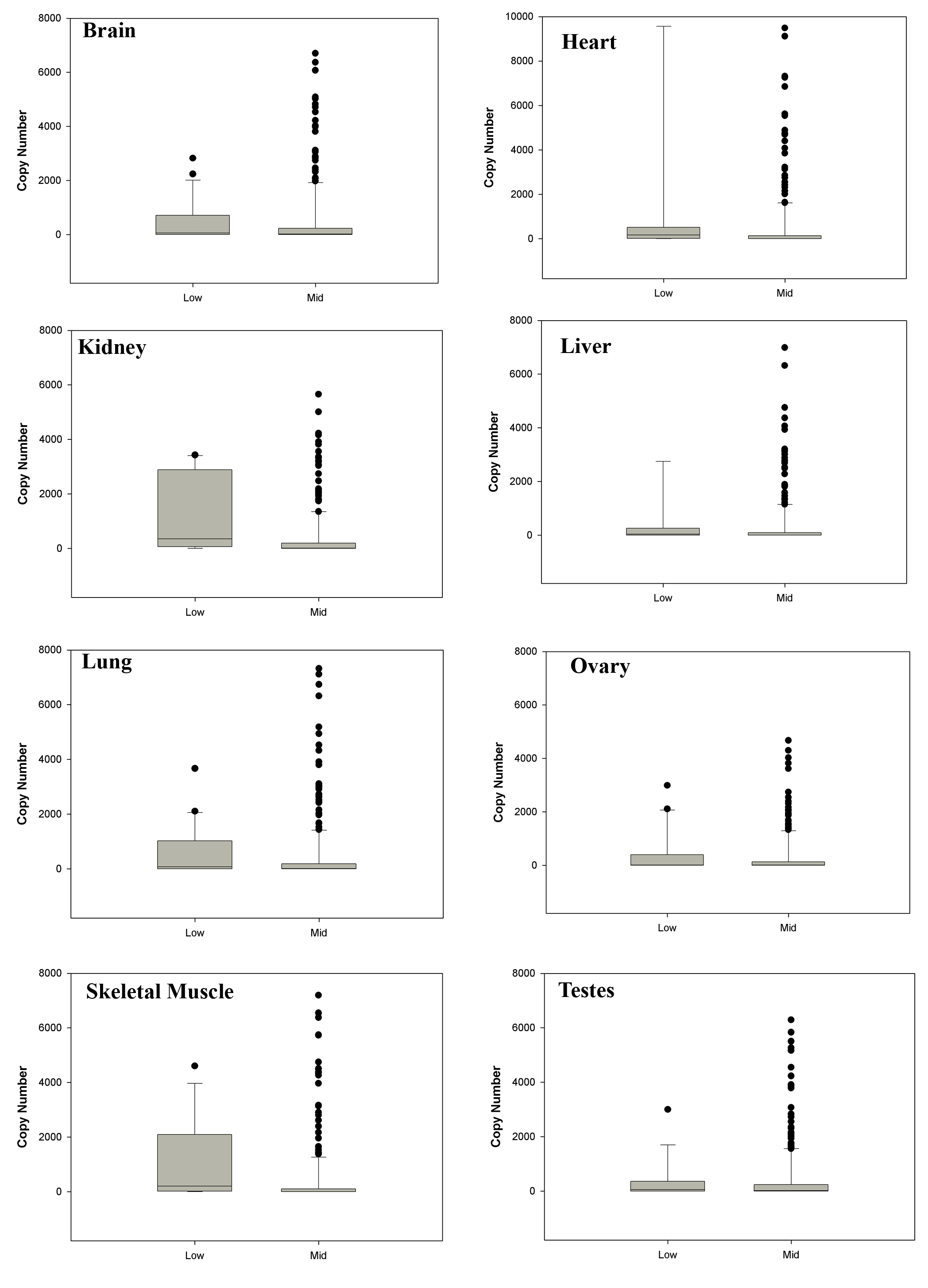

Supplement: Additional data file 10 — miRNAs were divided into groups according to gene expression of targets predicted from potential 5' UTR miRNA binding sites. For each tissue (brain, heart, kidney, liver, lung, ovary, skeletal muscle and testes) a box plot shows the miRNA copy number, as determined by RT-PCR [34] (y-axis) of the miRNAs in each group ('low' and 'mid', x-axis). [file gb-2008-9-5-r82-S10.tiff]
